# Assessing the implications of organised illegal and informal mining activities on the environment in South Africa

**DOI:** 10.1007/s13280-025-02251-4

**Published:** 2025-09-25

**Authors:** Richard Kwame Adom, Mulala Danny Simatele

**Affiliations:** School of Geography, Archaeology and Environmental Studies, University of Witwatersrand, Johannesburg, 2050 South Africa

**Keywords:** Environmental degradation, Illegal mining operations, Informal mining activities, Organised crime and mineral extraction, Resource governance

## Abstract

Organised illegal and informal mining is growing globally, employing many unemployed youth, women, and children. In South Africa, rural communities see it as a means to reduce poverty and integrate into the economy. Despite numerous laws and regulations by the government and the Chamber of Mines to curb these practices due to their significant environmental implications and health risks the problems are escalating instead. Furthermore, while some levels of success have been achieved in addressing these menace, the efforts by the government through legislation have failed considerably in addressing the environmental challenges associated with these activities. This study uses qualitative methods comprising of interviews, field observations, and literature materials to explore the root causes of these activities and their threat to environmental sustainability. The findings reveal poverty and unemployment as primary drivers, while weak governance and law enforcement as underlying factors. The study calls for a shift from punitive policies to inclusive strategies that address social and economic needs, stressing on education, awareness, and public participation in environmental challenges associated with illegal mining.

**Supplementary Information:**

The online version contains supplementary material available at 10.1007/s13280-025-02251-4.

## Introduction

Organised illegal and informal mining activities in South Africa, locally referred to as "Zama-Zamas", which means "those who try their luck", have become the most discussed issue in the country due to its exponential growth in many provinces across the country, Africa and Latin America (Mulaba-Bafubiandi and Mamba 2009). Stacey et al. (2025) stated that organised illegal mining operations are large scale, well-financed and coordinated operations that function without legal permits and often linked to criminal networks, corruption, and environmental degradation. While informal mining activities typically small-scale and labour-intensive, carried out by individuals or communities without formal regulations or licenses, driven largely by poverty and lack of alternative livelihoods and (Mkhize 2017). The rise in organised illegal and informal mining activities has opened up contentious debates around the world about communities and marginalised groups sharing their country's mineral wealth to alleviate poverty, enhance livelihoods and promote local communities to participate in the economies of their countries (Chelin and Els 2021). Similarly, the International Labour Organisation (ILO 2017) posited that over 13 million people are involved in the informal and organised illegal mining activities globally, and of that number, four million are in Sub-Saharan Africa. Isung et al. (2021) revealed that gold and gemstones extracted by informal and illegal mining undertakings had contributed over $1 billion annually to countries in sub-Saharan Africa, $200 million to China in 2018, $180 million to Bolivia and Brazil in 2017, $140 million in Indonesia and $250 million in Peru between 2017 and 2018.

Informal and illegal mining in South Africa has contributed significantly to the growth of the country’s economy the South African Chamber of Mines (CMSA 2017). The sector contributes approximately 5% annually to the country's GDP and employs more than one million people (Matshusa and Leonard 2022). To support this assertion, Mphokane (2018: 10) disclosed that informal and illegal mining activities are critical to the economy of South Africa. The sector contributes R8 for every R100 produced by the national economy and employs one in every 40 working individuals, which accounts for 2.5% of the total labour force in the country, consequently contributing to economic growth and stability for the country (Phala et al. 2017). Furthermore, Zvarivadza (2018) stated that the contribution of informal and illegal mining activities to the economy is significant. It is projected that that sector contributes more than 15% of the country's total foreign revenues, with gold topping the table and providing livelihoods to many households (Mphokane 2018).

While organised illegal and informal mining activities contribute to the socio-economic advancement of the country and driver of livelihoods to several households, nonetheless, these activities leave significant environmental challenges, particularly the contamination of groundwater and surface water, deforestation, soil erosion, and pollution on the immediate environment and broader impacts for the global environment (Witchalls 2022). The informal and organised illegal mining activities have been linked directly to the devastation of arable lands and the environment, specifically in river systems, while water bodies have been severely polluted and some declared unfit for human and agriculture use (Mulaba-Bafubiandi and Mamba 2009). In South Africa, informal and organised illegal mining activities have thrived due to the abandonment of large, wide, deep and dangerous uncovered holes left by large scale and commercial mining on agricultural lands and riverbanks (Kayet et al. 2021; Adom et al. 2024). In addition, the cost of informal and organised illegal mining, both economically and environmentally, is massive. The unauthorised and unregulated nature of the industry dictates that all the proceeds gained from the activities are not taxed (Hilson et al. 2017). Estimate by the South African Chamber of Mines (SACM) revealed that illegal mining activities cost the country more than R7 billion yearly, mainly due to non-payment of taxes and royalties (Cuya et al. 2021). The environmental costs of these illegal activities have resulted in public outcry and tension as the destroyed lands polluted water bodies, health safety issues and social impacts brought by these activities often lead to sporadic clashes between the communities and the illegal miners often resulting to loss of life and properties (Martin 2019). Consequently, the SACM warns that the Zama-Zamas activities if not addressed would lead to acute water crisis, uncontrollable land degradation, catastrophic deforestation, massive soil erosion that will lead to civil unrest in future (Phala et al. 2017). Table 1 conceptualises the devastation of the informal and illegal mining activities in South Africa.

**Table 1:** Environmental implications of informal and illegal mining in South Africa. *Source* Department of Mineral Resources, 2017

Category	Impacts	Scale of devastation	The number of people impacted
Land and Biodiversity	Soil degradation and deforestation	13 000 hectares degraded annually	Approximately 2.5 million rural residents affected due to reduced agricultural productivity and loss of livelihoods
	Abandoned pits and erosion	6000^+^ unrehabilitated sites	Over 500 000 people at risk of injuries and fatalities from unrehabilitated pits
Water resources	Mercury and acid mine drainage (AMD)	Mercury pollution from informal gold mining affects 3.5% of South Africa’s river systems	Over 3 million people exposed to contaminated water, especially in Mpumalanga, Gauteng and Limpopo provinces
Air pollution	Dust pollution and mercury emissions	10 tons of mercury emitted annually	Approximately, 1.2 million suffer respiratory issue
	Health impacts from air contamination	PM10/PM2.5 exceed safe limits by 50–150%	500 000 exposed to toxic emissions

Considering these complex challenges, many researchers, commentators, policymakers and experts in the sector have extensively researched into these issues and proposed strategies to tackle these critical and threatening crises. Phala et al. (2017), for instance, investigated the reclaiming of devastated lands brought about by organised illegal and informal mining activities with emphasis on restoration processes, strategies and costs involved. Mphokane (2018) evaluated the broadened the horizon of knowledge on informal and organised illegal mining by looking at how local communities will accept the challenge and responsibility of maintaining degraded landscapes as defector owners and prime beneficiaries of natural resources within the landscape. Brown (2017) evaluated the economic and social implications of illegal and informal mining activities on South Africa's economy. These authors concluded that informal and organised illegal mining undertakings contribute significantly to severe environmental and health-related complications and threats to the country's social, economic and environmental development that require urgent attention. While Hilson and Maconachie (2019) investigated work on artisanal mining in Africa, critically examined socio-economic dimensions and environmental impacts of small-scale mining, highlighting its role in rural livelihoods, the governance challenges it presents, and the need for formalisation and sustainable policy interventions to support vulnerable mining communities. However, what is missing in the various literature is the role of international syndicates and fraudsters in the spread of illicit mining activities and the severity of these activities on the environment. Considering these gaps, this paper investigated the following sub-heading: (a) underlying and primary causes of informal and organised illegal mining activities in Gauteng and Free State Provinces, (b) implications of organised illegal and informal mining operations on the environment and, (c) strategies to minimise the operations of informal and illegal mining operations on the environment. The rest of the paper is structured as follows: Section "Materials and methods" provides a literature-based overview of illegal mining activities in South African contexts and the implications of illegal mining undertakings on the environment. Section "Results" provides the materials and methodology of the paper, sections four deals with the empirical evidence and sections "Conclusion" and 6 look into discussions, conclusions and recommendations of the study.

While this study focuses on South Africa, however, the findings of the study will be of great benefit to other countries in the Sub-Saharan Africa particularly Ghana, the Democratic Republic of Congo (DRC), Zambia, Tanzania and many other nations. The findings will offer critical insights into the environmental, socio-economic, and governance challenges posed by organised illegal and informal mining activities. The recommendations will provide effective regulation, law enforcement, and policy reforms to institutions such as the Department of Mineral Resources and Energy (DMRE), the Department of Forestry, Fisheries and the Environment (DFFE), the South African Police Service (SAPS), the South African Human Rights Commission (SAHRC), and regional development partners such as the African Development Bank, and African Minerals Development Centre.

### Theoretical framework—Root causes of informal and illegal mining operation in South Africa

**Fig. 1:**
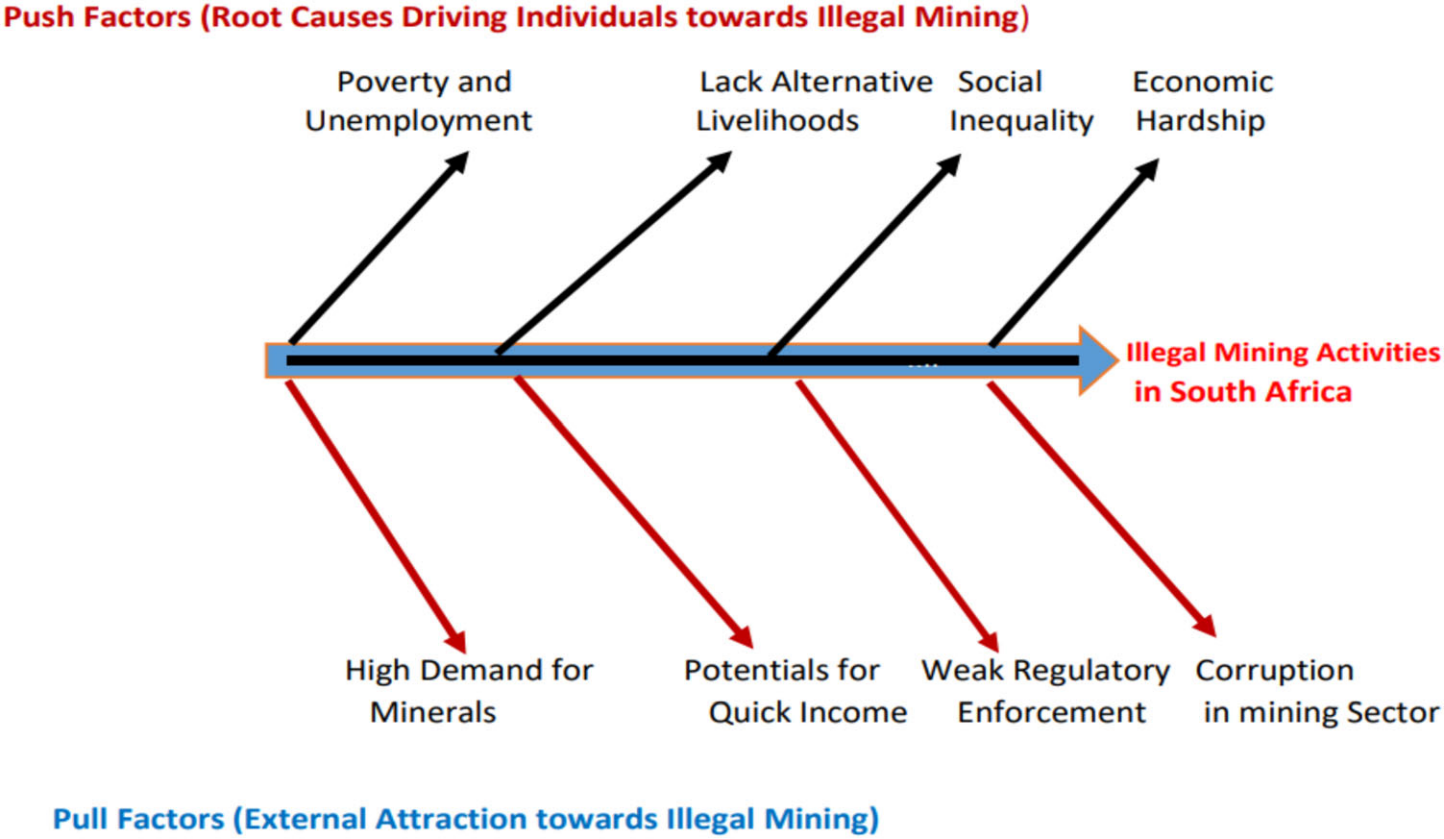
Conceptual framework of the causes of illegal mining operation

In the last ten years, empirical literature shows that South Africa is among the most lucrative countries for illicit minerals globally (Berman et al. 2017). Similarly, Phala et al. (2017) expressed that organised illegal and informal mining operations are a huge and growing problem across most of the provinces in the country (Fig. 10.1007/s13280-025-02251-4). The illegal mining operations are expanding and becoming complex, sophisticated and advanced. Many scholars and commentators, including Wilson et al. (2015), Mkhize (2017) and Osumanu (2020), identified two main factors contributing to the escalation of these activities in the country; these include "push and pull factors". Figure 1 conceptualises the push and pull drivers in a “fish bone” theory framework.

The push factors, according to Antwi-Boateng and Akudugu (2021), are root causes driving individuals into illegal mining activities in South Africa. Egunyu and Boakye-Danquah (2024) disclosed that conditional dynamics within neighbouring countries such as Zimbabwe, Lesotho, Mozambique and Malawi push some desperate people to South Africa, contributing significantly to the organised illegal and informal mining operations in the country. Nonetheless, Makhata and Masango (2021) mentioned that push factors such as loss of formal employment in the mining industry and lack of alternative employment opportunities among the local population, diminished agricultural land or agricultural productivity, declined profitability in businesses, high cost of living in the country, natural disasters and climate extremes, social inequalities and economic hardship contribute massively to illegal mining activities in the country.

On the other hand, Baddianaah et al. (2022) argued that pull factors attracting people into these illicit activities are lucrative. Egunyu and Boakye-Danquah (2024) identified ease of entry and high returns, receptivity, weak institutional capacity, corruption, willing compradors and existing social networks in the country and beyond. Similarly, Hentschel et al. (2003) further identified some of the pull factors as weak management of mines, easy access to mines, corruption, weak capacity and institutions, lack of resources, insufficient training, and judicial inefficiency, which hinder effective regulations. Cardwell et al. (2021) further expressed that the presence of available markets, driven by high demand for minerals, established black markets, and the integration of illegally mined materials into global supply chains, provide lucrative opportunities for illegal mining activities in the country.

## Materials and methods

This paper investigated the impact of informal and organised illegal mining activities in South Africa. It was carried out through a comprehensive study of the relevant literature and empirical research based on qualitative data (Fig 10.1007/s13280-025-02251-4).

### Research design

This research utilised exclusively qualitative approach comprising interviews, observation technique and secondary materials to explore the impacts of informal and organised illegal mining activities on the environment in Gauteng Province, Free State Provinces and by extension South Africa. A Semi-structured interviews with key stakeholders including environmental officials, local community members, law enforcement personnel, and representatives from civil society organisations served as the primary data collection method. These provided diverse perspectives on the immediate and underlying drivers, impacts, and governance challenges of the illegal mining activities in the country. To complement the interviews, secondary materials were engaged extensively to uncovere the relevant environmental legislation, enforcement strategies, and implementation challenges of regulations designed to address illegal mining operations in South Africa. This mixed approach offered contextual grounding and aided in triangulating the findings. Specific materials such as Mineral and Petroleum Resources Development Act (MPRDA), the General Laws Amendment Bill of 2024 that criminalised illegal mining and expanded law enforcement authority were extensively used. Others documents such as Draft Artisanal and Informal Mining (ASM), DMRE Policy Documents of 2021 were also utilised to obtained data on legislation framework of mining operations in the country. Additional documents such as Government Gazette No. 35265 of 2011 and Gauteng Gazette No. 295 of 2018 regulating suspension and revocation of mining rights, abandonment and non-compliant sites were also engaged extensively. Other important secondary materials engaged include the 2021 Parliamentary Committees’ reports on mining, news documentaries of the October 2022 on rehabilitation programs as well the Welkom mine explosion of 2023 and the Stilfontein standoff of 2024 coverage on the life and danger of underground illegal mining operations in South Africa. These memoires were widely engaged to obtained illicit dealings in the informal mining activities, legal framework, enforcement actions, revoked mining rights, policy responses, economic impacts, and the role of organised crime in driving illicit mining activities.

In addition to the above, participant observation at affected sites were carried out to augment and validate data obtained from the interviews and secondary sources. The observations were carried out to capture on-the-ground environmental conditions, informal activities, and community dynamics that might not be fully revealed through interviews alone. The field observations were conducted exclusively in the informal settlements surrounding disused mine shafts in area around Welkom in Free State and Benoni, Boksburg and Krugersdorp in Johannesburg. The fieldwork took place over six months (January–June 2023), focussing on environmental degradation, hazardous mining practices, and associated community impacts. Key subjects included informal miners, degraded landscapes, water bodies pollution, and the threats of these activities on nearby residential areas. Data collection instruments included structured observation checklists, a digital camera for photographic documentation, and field journals for note-taking. Photographs captured ethically with consent were used analytically to complement and validate interview responses, and visually illustrate patterns of environmental damage, unsafe working conditions, and the socio-economic consequences of illegal mining. Prior to the commencement of data collection, ethical clearance was diligently obtained from all relevant institutional bodies, including mining-related authorities, local municipalities, and community leadership structures. Given the sensitive and often illicit nature of informal and organised mining activities in South Africa, specific attention was paid to securing informed consent and ensuring participant confidentiality. Engagements were conducted in alignment with national research ethics guidelines, and approvals were sought where necessary from regulatory entities overseeing mining and community development.

### Sample population and size

A total of 20 respondents were purposely selected for face-to-face interviews. Purposive sampling is a non-random sampling technique where researchers select participants based on specific characteristics such as knowledge and experience relevant to the study's objectives and the ability to ensure that the most pertinent questions can be addressed (Campbell et al., 2020). This sampling technique ensured the inclusion of individuals or groups with critical insights and experiences related to illegal mining operations in Gauteng Province and South Africa in general. Specifically, two respondents each were picked from these institutions: South African Chamber of Mines and National and Gauteng's Department of Mineral and Resources (D.M.R.), Gauteng Department of Cooperative Governance and Traditional Affairs (COGTA), Department of Environment and Forestry and two senior lecturers from the Department of Mining Engineering of the University of Witwatersrand. Finally, a total of ten community leaders were intentionally selected from Benoni, Boksburg, Daveyton, Germiston and Krugersdorp. All the interviews were carried out between the period of June and November 2023.

The questions for each group were designed to elicit comprehensive and insightful responses, providing a multi-faceted understanding of the implications of informal and illegal mining in South Africa and Gauteng Province specifically. Precisely, questions related to exploring informal and illegal mining operations in South Africa, the economic impact, regulatory challenges, collaboration efforts, sustainability measures and policy recommendations were asked from the South African Chamber of Mines; the community impact, inter-departmental coordination, development programs, local government roles and successful intervention case studies were sought from the Department of Cooperative Governance; while the regulatory framework, enforcement strategies, licensing issues, data trends, and stakeholder engagement pursued from the Gauteng's Department of Mineral Resources (DMR); the research insights, theoretical perspectives, socio-economic factors, policy implications, and comparative analysis from academic lecturers. Finally, the community leaders provided valuable insights into the local impact of illegal mining, including economic, social, and environmental effects; community perceptions; safety concerns; economic dependence; conflicts and tensions; local responses and actions; support and resources available; perspectives on sustainable alternatives; feedback on policies; and examples of successful collaborations with other stakeholders. Due to the highly sensitive nature of illegal mining operations, it was imperative that explicit permission was obtained prior to conducting any data collection activities. This precaution ensures the protection of all involved parties and compliance with legal and ethical standards. The duration of each interview depended on the interviewee's status and the information being sought; however, an average of 30–60 min was given to each interviewee.

### Data analysis

Data obtained were analysed using a thematic approach. A thematic approach is a method of analysing qualitative data that involves identifying, organising, and interpreting patterns or themes within the data. It allows researchers to systematically code and categorise information to uncover insights related to participants' experiences, perceptions, and behaviours (Kiger and Varpio 2020). In process of analysing the interviewees responses obtained, firstly, we transcribed verbatim and anonymised the data to ensure confidentiality. The research team engaged in repeated readings of the transcripts to gain familiarity with the content before proceeding to thematic analysis using a hybrid approach. Open coding was prior conducted to identify key concepts and repeated ideas across participant responses. The codes were then grouped into broader categories through axial coding, followed by selective coding to derive overarching themes. Codes such as ‘livelihood survival’, ‘youth unemployment’, ‘environmental degradation,’ and ‘institutional inaction’ informed the development of themes including ‘mining as economic necessity,’ ‘governance and regulatory gaps,’ and ‘community perceptions of legality.’ These themes were grounded in participant narratives and aligned with the study’s research objectives. A codebook was maintained to ensure consistency, and findings were reported with illustrative quotes to capture the diversity of perspectives among stakeholders.

## Results

The data obtained from the field survey were organised and structured based on the objectives set up for the study, which include the causes of informal and illegal mining activities, the implications of the mining activities on the environment and strategies for coordination and improving these mining activities without harming the environment.

### Causes of organised illegal and informal mining activities in South Africa

Identifying the root causes of informal illegal mining endeavours is a fundamental driving force for this study. Given these reasons, we explored these issues with respondents on the field. The comments below reflect the views of respondents engaged. A Director of the Gauteng Department of Environment and Forestry who was engaged in an interview disclosed that:

"The growth in informal and illegal mining is now taking place on a large scale in the province and nationally, could be attributed to the combination of difficult social and economic factors such as high levels of unemployment, rising poverty, limited resources, cumbersome procedures to obtained mining licence to be registered mining activities" Per.com 2024 1A.

Contrary, a respondent from the South African Chamber of Mines who was contacted to share her view on this matter stated that:

"Poverty and unemployment are not the fundamental causes of illegal mining activities in the country. She holds the view that the syndicates and kingpins of these activities are not poor; these kingpins are just capitalising on the strong demand for the minerals both at home and abroad. According to this interviewee, the illegal miners and their operations had often resulted in worse conditions after the operations concluded than before they began the illicit activities" Per.com 2024 1B.

The interviewees’ views affirmed the push–pull model and fishbone theory. The former highlights how poverty, unemployment, and weak enforcement drive illegal mining, while the latter identifies root causes of environmental harm—policy gaps, institutional failures, and poor land use. Together, they reinforce the thematic analysis and clarify the socio-environmental impacts shown in Fig. 1.

### Impacts of organised illegal and informal mining activities on the environment

This paper analysed extensively the impact of informal mining on the environment, namely, water, land and air pollution in South Africa. Respondents were asked during interviews to enumerate the implications of these activities on water resources. Views and comments of respondents are presented below. A Director from the National Department of Environment and Forestry stated in an interview:

"Informal and illegal mining generates vast wastewater and tailings, leading to serious water pollution. This interviewee noted that mine water is often contaminated and rarely treated before discharge. He identified four major pollution types from mining: acid mine drainage, heavy metals, chemical pollutants, and sedimentation, highlighting the urgent need for proper environmental safeguards" Per.com 2024 1C.

Another respondent from the community also mentioned that:

"Mining operations routinely modify the surrounding landscape by exposing previously undisturbed earthen materials. Erosion of exposed soils extracted mineral ores, tailings, and fine material in waste rock piles can result in substantial sediment loading to surface waters and drainage ways. In addition, spills and leaks of hazardous materials and the deposition of contaminated wind" Per.com 2024 1D.

In support of participant claims about river pollution, field observations at the Ga-Sekhukhune site revealed visible siltation and discolouration of the Klip River, with stagnant pools of muddy water downstream from mining activities. This corroborates respondents’ concerns about the contamination of water sources used for household consumption and farming.

The research team obtained data from relevant institutions and communities through interviews regarding the implications of mining activities on land. The perspectives of respondents are presented below. An employee in the South African Chamber of Mines expressed this when approached for an interview:

Deforestation is a major part of the damage Zama-Zamas causes to the land. It involves clearing the forest, leading to the cutting down of trees, to enable them to extract the minerals. These illegal pit workers also do not put in place any measures to safeguard them from risk. The fact that illegal sappers are mostly unskilled; they also use unprotected tools and equipment, making them a big threat to the nation. Per.com 2024 1E.

This was buttressed by a Director of the South African Chamber of Mines, whom we approached for Her opinion on the subject matter. This interviewee stated:

"Gauteng's mining towns, normally poor and informal, are impacted the most by these mining activities. Neglected mountains of sandy dirt from the mining sites are ladened with significant particles of arsenic materials and other dangerous chemicals. Through wind blows, contaminated dust is blown into nearby homes, where it settles on people's roofs, roads, schools and even where children play playgrounds. This dust gets into our food; we eat this dust, we drink this dust, and these are the reasons why most people in the province get sick. This is a silent killer" Per.com 2024 1 F.

Environmental Impact Assessment (EIA) reports obtained from the Department of Mineral Resources (DMR) for the region show that illegal mining sites often fall outside officially designated areas and bypass environmental compliance processes. This supports interviewees’ accounts of unregulated mining posing greater ecological risks than licensed operations.

### Way forward to make mining more environmentally friendly

Identifying strategies to suppress illegal mining and improving the mechanisms of informal mining forms an integral component of this study. In the light of this, we explored the views and perspectives of the respondents on this matter. Suggestions and proposals obtained in the field are presented below. Interaction with the Provincial Director of Gauteng's Department of Mineral Resources stated that:

"An interviewee stressed the urgent need to close South Africa’s illegal and unregulated mining sites through strict enforcement. Citing China’s 2010 response to reckless mining with its Rare Earth Industrial Development Policy, they argued that firm government action can set a powerful environmental precedent, promote greener practices, and align the sector with sustainable and responsible resource management" Per.com 2024 1G.

A member of a community in the Krugersdorp in the Gauteng Province proposed during the one-on-one interview that:

"Green mining education, including technical, environmental training and information, will increase expertise, capacity and know-how to improve informal mining and minimise environmental degradation that comes with mining activities. In South Africa today, the youth and women face multiple, interconnected barriers that restrict their ability to own mining licences and permits to undertake legitimate mining activities. Resource allocation gaps make it extremely difficult to be inculcated into the mainstream economy and live a meaningful life " Per.com 2024 HI.

The Gauteng Gazette No. 295 of 2018 and Government Gazette No. 35265 of 2011 highlight the legislative gaps in enforcing informal mining controls. These policy documents reflect the institutional weaknesses mentioned by key informants and substantiate the claim that fragmented governance contributes to unchecked environmental harm.

## Discussion

The discussion of this paper is guided by objectives set out by the researchers, which include identifying the causes of organised illegal and informal mining activities in South Africa, the implications of the activities on the environment and strategies for suppressing illicit activities and improving strategies for informal mining undertakings in the country.

### The primary and underlying causes of organised illegal and informal mining activities

This study explores the multi-faceted causes and consequences of organised illegal and informal mining activities in South Africa particularly in the Gauteng and Free State provinces. Drawing on field observations, interviews, and scholarly work including Ledwaba (2017), Agustina et al. (2021), Tracy-Lynn (2022) and Arthur-Holmes et al. (2023), it was uncovered that organised and informal mining practices are driven by a combination of push and pull factors as shown in Fig. 1. The key findings from our engagement revealed that push factors are widespread which are driven mainly by high levels of unemployment, poverty, lack of alternative livelihoods, as well as absence of effective policies and programme that integrate marginalised communities into the formal economy. Our engagements revealed that socio-economic hardships pertaining in the neighbouring countries of South Africa particularly, Lesotho, Malawi, Zimbabwe, and Mozambique, are driving individuals to migrate and engage in illegal mining activities in South Africa (Table 10.1007/s13280-025-02251-4). Pull factors on the other hand, include the relatively easy access to mineral-rich lands, informal networks and social capital supporting illegal operations, and the promise of steady income. The study utilises the push–pull framework and the fishbone model to explain how these variables interact. The push–pull approach demonstrates how socio-economic exclusion drives individuals towards illegal mining, while the fishbone model maps the broader systemic issues and environmental consequences, such as land degradation, water pollution, and deforestation. This is demonstrated in Fig. 1 and Fig. 10.1007/s13280-025-02251-4 in the literature review.

However, our research goes beyond these traditional explanations by identifying deeper structural and underlying drivers to the organised mining operations in the country. Our findings uncovered that the underlying driver to these activities are the presence of unrehabilitated, and abandoned mines, which provide opportunities for illegal operations. Furthermore, the existence of lucrative domestic and international markets for illegally mined resources, combined with weak enforcement mechanisms and under-resourced security and immigration authorities are fuelling the problem. Corruption among key actors in the mining sector, including officials and law enforcement are facilitating the growth of criminal networks involved in these activities. The findings resonate with prior research by Phala et al. (2017) that links illegal mining to global organised crime. These operations are often managed by sophisticated, well-financed syndicates capable of evading or overpowering even the most stringent security systems. Revenues from illegal mining are used to fund conflicts in fragile states such as the D.R.C., Colombia, and Afghanistan, with implications for global security and governance. Legal and institutional shortcomings are also playing a critical role. The underling findings of from some experts engaged revealed that he Mineral and Petroleum Resources Development Act (MPRDA) and similar laws enforcers across the country lack the legal clarity and enforcement capacity needed to regulate the sector. The governance model is often top-down, excluding local communities from decision-making and benefits. This exclusion contributes to the rise of heavily armed, violent mining groups that pose serious threats to national security. A noteworthy finding from the field observation revealed that the environmental and social implications from these activities are profound. Rather than improving livelihoods, illegal mining depletes agricultural land, contaminates water sources, and undermines food security. It contributes to poverty, displacement, occupational health hazards, and increased violence, including xenophobic attacks.

### Environmental implications linked to organised illegal and informal mining activities

**Fig. 2:**
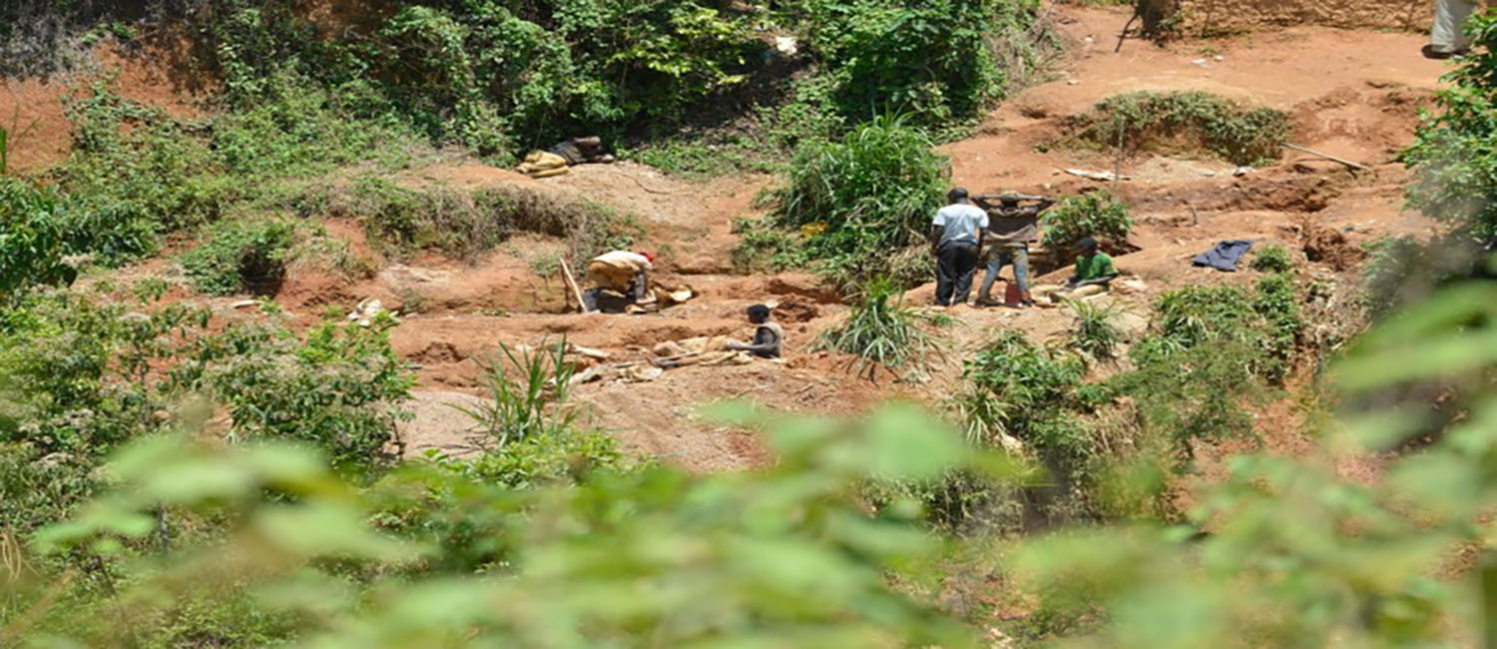
A picture of the devastation in the Benoni community in Gauteng by illegal mining activities. **Source:** A field observation by the Research Team September 2023

The environmental impacts of organised illegal and informal mining in South Africa particularly in areas such as Benoni in Gauteng Province are profound. Interviews, field observations, and secondary sources consistently show that miners employ unsustainable methods, such as surface and underground excavation without post-mining rehabilitation. Because they lack modern machinery, informal miners dig numerous shallow pits across large areas, leaving behind unfilled holes that degrade the land and disrupt ecosystems. Field evidence from Benoni shows extensive land degradation, deforestation, and soil erosion near unregulated mining sites (Fig. 2).

**Fig. 3:**
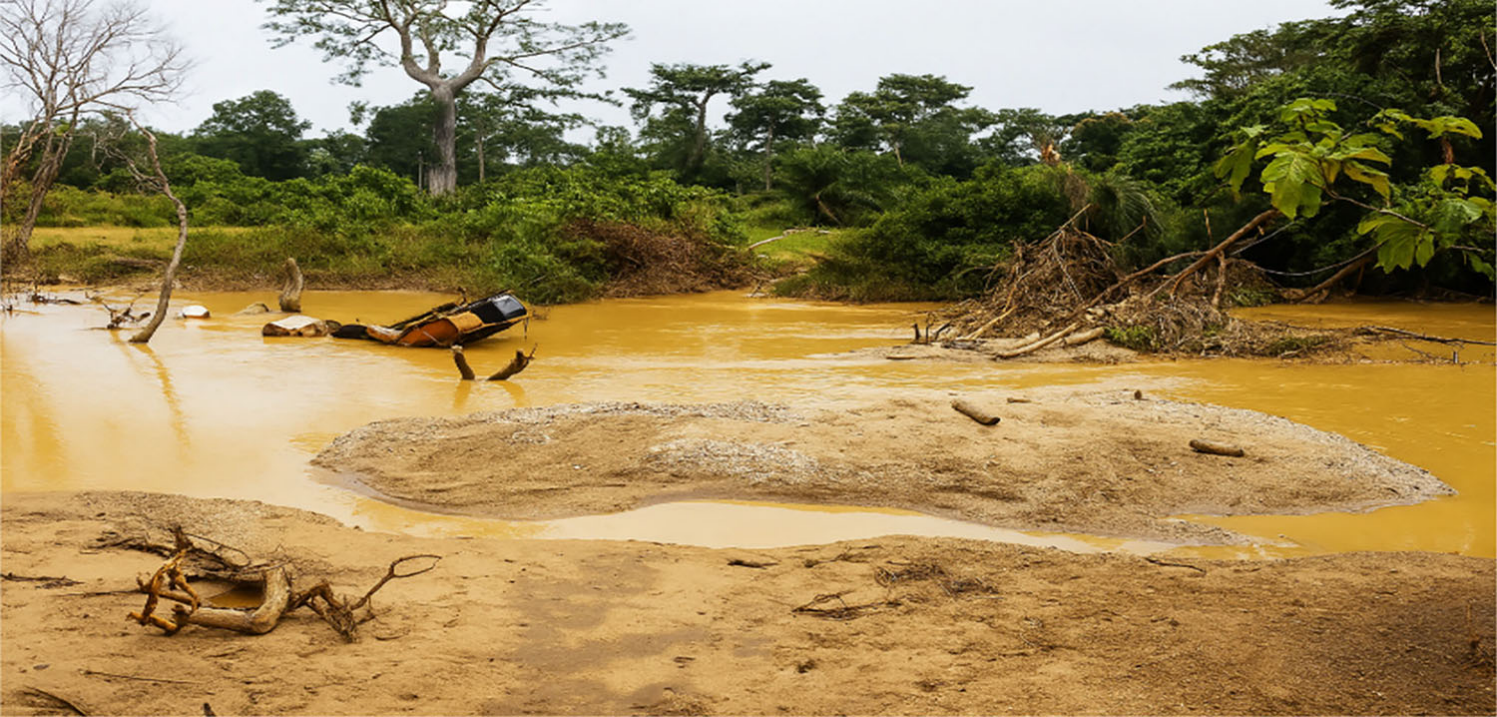
The consequences of illegal mining on Klip River in Gauteng Province. **Source:** A field observation of the Research Team September 2023

These findings align with previous studies (Haddaway et al. 2019; Obeng et al. 2019; Bansah et al. 2023) and South Africa's 2021 Parliamentary Report, which link these practices to biodiversity loss, water contamination, and habitat destruction. One major environmental concern is water pollution, where mining operations contaminate rivers, dams, and streams with sediments, oils, and toxic chemicals like mercury. This not only endangers aquatic life but also renders water unsafe for agriculture, households, and industrial use. A case in point is the Klip River in Gauteng, polluted by chemical drainage and oil spills from nearby mining activities shown in Fig. 3.

Additionally, our engagements with experts and observation from the field revealed that waste discharges from informal mining, particularly during rainfall, accelerate soil erosion and cause severe siltation in waterways. This affects water quality, aquatic habitats, and increases the risk of flooding and water scarcity for downstream communities (Table 10.1007/s13280-025-02251-4). These findings are also expressed by Attiogbe and Nkansah (2017) and DMRE (2021) that illegal mining activities are contributing to increasing acidity and pH imbalances, harming organisms that dependent on these resources and reducing access to potable water and fish stocks. These activities are also impacting significantly on land use and agricultural productivity. We observed that these operations are creating large-scale soil displacement, deforestation, disrupting ecosystems, and destroying arable land. Furthermore, our engagements and field observations uncovered that most of the informal mining sites are littered with open pits, waste rock piles, and subsided land, threatening infrastructure such as roads and buildings. The removal of topsoil and deep excavations destabilise the land, raising risks of sinkholes and structural collapse. This not only deters future development but undermines local economies and increases vulnerability in already under-resourced provinces.

Additionally, it was established through the field observations that these mining activities are modifying the landscapes, destroying flora and fauna, and altering the cultural and ecological heritage thus, reducing the viability of tourism businesses; a livelihood source for many. These outcomes are echoed by Arthur-Holmes et al. (2023), that the illegal mining operations are contributing significantly to loss of habitats across the biological spectrum. Air pollution is another serious consequence associated with these activities. Our observations revealed that open-pit and illegal mining activities are releasing fine particulates (PM_10_, PM_2.5_), toxic gases (SO_2_, NO_*x*_), and heavy metal-laden dust from drilling, blasting, and fuel combustion. Such emissions are linked to respiratory and cardiovascular diseases, ecosystem degradation, and regional air quality decline. Similar findings are (Singh 2022; Kudjoe et al. 2019) that exploration and operational phases of illegal mining are particularly harmful, contributing to diseases such as silicosis, asbestosis, and lung cancer.

### Improving mining strategies of informal and illegal for environmental sustainability

Given the profound environmental challenges posed by organised illegal and informal mining activities in South Africa (Tab. 10.1007/s13280-025-02251-4) particularly, Gauteng and Free State Provinces, this study explored alternative operational strategies aimed at minimising the ecological damages, with a focus on promoting green mining practices. Data were drawn from interviews, field observations, and secondary sources. A key consensus from these engagements is that eradicating illegal and informal mining entirely is unfeasible due to entrenched socio-economic hardships in South Africa and neighbouring countries. However, stakeholders including community leaders, government officials, and parliamentary reports agreed that the government and key sector players must adopt policies that mitigate environmental harm and promote eco-friendly operations. Findings indicate that curbing illegal mining requires a dual strategy: confronting the immediate threat while addressing underlying socio-economic drivers. Interviewees, supported by the DMRE Policy Documents (2021) and field observations, consistently identified poverty, unemployment, and lack of alternatives as major push factors. In all studied mining communities (Benoni, Boksburg, Daveyton, Germiston, Krugersdorp, and Welkom), illegal mining was found to thrive in conditions marked by limited education, scarce employment, and weak institutional support. Investing in sustainable alternative livelihoods such as vocational training, small-scale enterprise development, agro-processing, and green economy jobs was widely recommended. Such interventions would reduce economic incentives for illicit mining while fostering social inclusion and long-term environmental stewardship.

These findings aligned with scholars such as Arthur-Holmes et al. (2023), and Hilton et al. (2020), who argue that reducing illegal mining globally requires radical economic reforms that absorb unemployed youth into formal labour markets. Similarly, Phala et al. (2017) advocate for regional cooperation among neighbouring countries to address poverty and unemployment, thereby limiting illegal migration and its mining-related consequences. Another recurring theme in interviews and government reports, including those from Parliament’s Portfolio Committee and the DMRE, is the link between abandoned, unsealed mining shafts and illegal mining activities. In hotspots like Benoni, Krugersdorp, and Welkom, unsecured and poorly decommissioned mines serve as operational bases for illicit miners. These sites lack necessary physical barriers, monitoring systems, and rehabilitation efforts, enabling criminal syndicates to entrench themselves. The failure to properly close and secure these sites not only perpetuates illegal mining but also poses environmental and public safety risks. The underlying views from experts suggest a multi-tiered strategy is required to rehabilitate and monitor abandoned sites. These involve environmental authorities, law enforcement, and local communities. These findings are also shared by Matshusa and Leonard (2022) and Mhlongo (2023), who emphasised the need for effective legislation and funding mechanisms to ensure that mining companies take responsibility for proper closure and environmental restoration.

In addition to land rehabilitation, our engagements in the communities such as Welkom and Benoni suggest the need for a specialised, multi-agency task force equipped with intelligence and enforcement capabilities to dismantle illegal mining networks. Similar views are supported by Handelsman (2002), Arthur-Holmes et al. (2023), and Mkhize (2017), that the current police forces are often under-resourced and ill-equipped to confront heavily armed and well-organised mining syndicates. Lastly, government representatives and community stakeholders, along with policy documents like the MPRDA (Act No. 28 of 2002) and DMRE’s Guideline for Rehabilitation of Mined Land, stressed on improving environmental performance. Illegal mining disturbs natural processes, causing long-term damage far beyond that of natural erosion. We propose systematic assessment and mitigation strategies to manage environmental impacts. This includes investing in eco-friendly waste disposal systems, promoting waste-to-resource technologies, and enforcing regular restoration of mined land to ensure communities remain habitable post-closure. These findings reflect Maddala et al. (2021), who advocate for green mining practices such as clean technologies, community involvement, and sustainable supply chains.

## Conclusion

Informal and illegal mining activities in South Africa represent a complex challenge with far-reaching environmental consequences. Predominantly driven by poverty, unemployment, and exclusion from the formal economy. These illicit activities contribute severe to land degradation, water and air pollution, biodiversity loss, and increased criminal activities particularly in mining communities. While the Department of Mineral Resources and Energy and the South African Chamber of Mines recognise informal mining as a legal activity, most operators lack licenses due to the high costs and bureaucratic hurdles, rendering many of them illegal. Illegal mining, though prohibited, continues to rise, involving both citizens and foreign nationals who support operations with resources and financing. Undoubtedly, the rise of these activities have contributed to significant environmental challenges in the form of deforestation, soil erosion, siltation and soil compaction, destruction of the ecosystem and loss of biodiversity, as well as substantial impacts on the country's water, and air resources through the release of toxic chemicals and pollution, resulting from directing and indirect impacts on local communities through forced displacements, floods, health complications and human right violations. While everyone agrees that the problems brought about by illegal and informal mining need to be addressed, the question of how seems to be on the back burner.

To address these, menace this study recommends a transitioning from criminalization to formalisation through a developmental and inclusive regulatory approach that includes accessible licensing frameworks, participatory Environmental Impact Assessments (EIAs), community-based environmental monitoring initiatives, technical and financial support for sustainable mining practices, and multi-stakeholder governance platforms that bring together government, civil society, traditional leaders, and miners to ensure accountability, environmental protection, and socio-economic development. Furthermore, it is imperative to recognise the historical role of informal mining during colonial and apartheid eras, policy reforms must legitimise and redefine small-scale mining to benefit marginalised populations while supporting national revenue generation. The state should classify mining within a defined threshold as informal and provide access to capital, geological data, and equipment.

The legislative framework in mining must address social and economic realities, focussing on six principles: legal reform, access to resources, capacity building, and multi-stakeholder dialogue. Our study established that informal miners predominantly use rudimentary techniques such as open-pit and underground mining without environmental assessments, severely impacting ecosystems. Due to limited education and awareness, environmental damage is often unrecognised. This study strongly recommend that the government promote green mining by subsidising cleaner technologies, adopting advanced land rehabilitation methods, while educating miners and the public on environmental risks through media campaigns, schools, and community platforms. Mineral extraction under these informal systems have resulted to habitat destruction, vegetation loss, topsoil erosion, and water contamination. To restore ecosystems, urgent reclamation and rehabilitation strategies such as reforestation and water diversion are necessary. Additionally, stricter but community-inclusive regulations are needed to ensure that any expansion of mining benefits local populations and mitigates environmental damage. While zero impact may be unattainable, improved policies and stakeholder engagement can significantly reduce the harms.

Due to time and resource constraints, the study could not address all dimensions of the issue. Future research should explore transnational networks fuelling the illicit mineral trade, protection structures contributing to illegal operations, and technological tools used by syndicates. Longitudinal studies are also needed to assess the long-term effects of these practices.

## Supplementary Information

Below is the link to the electronic supplementary material.

Supplementary file 1 (DOCX 1247 KB)

## Data Availability

The data supporting the finding of this study are available on request from the corresponding author. The data are not publicly available due to privacy and ethical restriction.
