# Supplementary material for: Assessing the implications of organised illegal and informal mining activities on the environment in South Africa
Source: Ambio. 2025 Sep 25;55(3):558–70. doi: 10.1007/s13280-025-02251-4 (PMC12868526; doi:10.1007/s13280-025-02251-4)
Supplement: Supplementary file 1 — (DOCX 1247 KB) [file 13280_2025_2251_MOESM1_ESM.pdf]

Supplementary Materials

Richard Kwame Adom [1*] Mulala Danny Simatele [1]

[1.] School of Geography, Archaeology and Environmental Studies, University of Witwatersrand, Johannesburg South Africa 2050; richardquame1@gmail.com, Richard.Adom@wits.ac.za

Mulala.Simatele@wits.ac.za

*Correspondence author (richardquame1@gmail.com)

Assessing the Implications of Organised Illegal and Informal Mining Activities on the Environment in South Africa

1. Supplementary literature review: The Organised Illegal and Informal Mining Operations Globally and in Sub-Saharan Africa: The Primary and Underlying Drivers of Informal and Illegal Mining Operations.

The organised illegal and informal mining activities in Sub-Saharan Africa and other developing countries are driven by a complex interplay of proximate and underlying factors, including economic, social, regulatory, environmental, technological and socio-political factors (Asori et al., 2023). Arthur-Holmes et al. (2022) posited that abject poverty, high unemployment levels and the immediate financial returns from these activities are the proximate or primary causes. Bansah et al. (2023), also expressed that social factors such as migration to urban centres, traditional practices, weak regulations, ambiguities in legislation, and misinterpretation of community-ownerships are some of the underlying drivers of the informal and organised illegal mining operations in most countries on the continent. Similarly, Afriye et al. (2023) argued that weak governance, poor regulatory frameworks, and corruption among state agencies are contributing to the rise of organised illegal the informal mining activities in Africa and other parts of the world. Supporting these perspectives, Arthur-Homes et al. (2022) expressed that ambiguous land tenure and easy accessibility of mineral deposits are driving these activities in some countries on the continent, particularly Ghana, Democratic Republic of Congo and Zambia. Other scholars and commentators such as Frederiksen (2019) and Scheidel et al. (2023) expressed that external factors such as global commodity prices, availability of markets both local and abroad, together with socio-political instability, push certain communities towards mining for survival. Moreover, Ofosu et al. (2023) disclosed that lower education levels limit formal employment opportunities, and cultural acceptance of mining into local traditional setups and lifestyles promotes these activities. Figure 1 simplifies the proximate causes and underlying drivers of organised illegal and informal mining activities in Africa.

Supplementary Figure (S1): Proximate causes and underlying drivers of informal and illegal mining activities in Africa

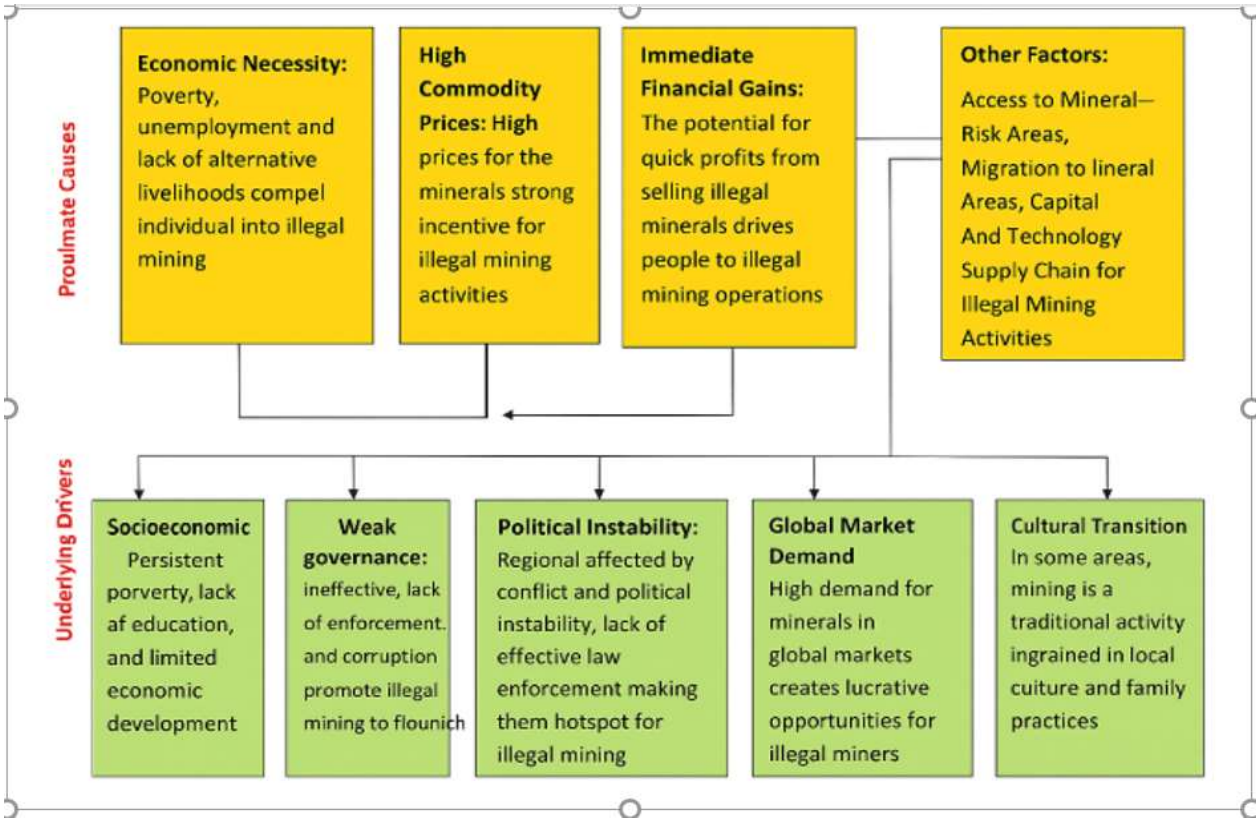

In spite of economic gains and livelihoods provided by these mining operations mostly to the informal sector across the continent and beyond, most of these countries, particularly Ghana, the Democratic Republic of Congo, Zimbabwe, Zambia, Tanzania and Nigeria, are experiencing the worst implications on their environment linked to organised illegal and informal mining operations (Kumah et al., 2022; Afriye et al.., 2023). Asuamah (2023) expressed that the unregulated nature of informal and illegal mining operations has contributed to severe deforestation, habitat destruction, water pollution, soil degradation, loss of biodiversity poses serious health risks on local communities, gangsters, kidnapping, violent and gang wars that sometimes result to loss of life and properties of innocent population. Nti et al. (2023) stated that in Ghana, illegal mining, popularly known as "galamsey," has resulted in the pollution of over 60% of the country's water

bodies, significantly increasing the cost of water treatment and affecting the availability of clean water. Furthermore, the country has experienced loss of approximately 25% of its forest cover between 2001 and 2021, with illegal mining being a major contributing factor (Kyere-Boateng et al., 2021 and Adom et al. 2024). Furthermore, Tuffour et al. (2024) disclosed that over 1.5 million hectares of the country's lands have been degraded due to open cast mining methods and harmful chemicals by illegal miners.

In the Democratic Republic of Congo (DRC), illegal mining has led to the annual destruction of over 1,500 square kilometres of its vast tropical rainforests, contributing to deforestation and habitat loss (McFarland et al., 2018). Mulenga et al. (2023) posited that water contamination from toxic substances like mercury and cyanide are posing severe risks to local communities and the aquatic ecosystem. Verweijen et al. (2022) stated that the country's rich biodiversity is under severe and constant threat due to the encroachment of informal and illegal mining operations. Similar conditions pertain in Zimbabwe, Zambia, and Nigeria (Oruonye et al., 2023; Madimu, 2022). Zimbabwe is currently facing significant environmental challenges due to illegal gold mining activities, which is causing severe river siltation and destroying water quality and availability (Jongwe et al., 2023). These authors stated that the Zimbabwe National Water Authority (ZINWA) report that heavy siltation in major rivers such as the Mazowe and Odzi have exceeded the global average of 25 milligrams per litre. The widespread use of mercury in gold extraction has led to mercury pollution, with levels in some areas exceeding World Health Organization (WHO) safety limits by up to 50 times (Soe et al., 2022). Similarly, illegal mining is causing severe soil erosion, currently, over 100,000 hectares of land has be degraded beyond cultivation (Moyo et al., 2022). In Zambia, illegal mining, particularly for minerals such as copper and cobalt, is contributing significantly to forest degradation, with an estimated annual loss of 300,000 hectares of forest (Day et al. 2014). Heavy metals from illegal mining activities have contributed to the contaminated of soils and have affected agricultural productivity (Day, et al., 2014). Similarly, Manissalidis et al. (2020) expressed that air pollution has also worsened due to illegal mining operations. These authors posited that increasing levels of particulate matter and toxic gases have exceeded national air quality standards in most of these mining communities in the country. Table 2, summarises the organised illegal and informal mining operation and its devastating consequences in some selected countries in the sub-Saharan Africa

(Supplementary Table (S1): Illegal mining operations in some countries in sub-Saharan Africa

Countries	Root Causes	Area of Land Degraded (Hectares)	Volume of Water Resources Destroyed (m^3)	Populated Affected
Ghana	Weak enforcement of mining laws, unemployment, and demand for gold and other minerals	30,000$^+$ hectares	55 million m^3 (e.g. River Pra & Ankobra)	1.1 million$^+$

Zimbabwe	Economic instability, lack of alternative livelihoods, and corruption in mineral governance	5,000+ hectares	12 million m^3	300,000$^+$
Democratic Republic of Congo	High demand for rare earth minerals (e.g., cobalt), weak governance, and armed conflict funding mining	20,000$^+$ hectares	30 million m^3	2 million$^+$
Nigeria	High poverty rates, lack of enforcement of artisanal mining laws, and demand for gold	10,000$^+$ hectares	15 million m^3 (e.g. Zamfara water bodies)	600,000$^+$
Zambia	Demand for copper and cobalt, weak oversight of artisanal mining, and high unemployment	8,000$^+$ hectares	20 million m^3 (e.g. Kafue River pollution)	400,000$^+$
Sierra Leone	Demand for diamonds, lack of regulation in artisanal mining, and high poverty levels	15,000$^+$ hectares	25 million m^3 (e.g., Sewa and Pampana Rivers)	700,000$^+$

Source: United Nations Environment Programme (UNEP), 2018

However, the informal and organised illegal mining operations are also experienced in other parts of the world, particularly in countries such as Brazil, Peru, Venezuela, Columbia, Bolivia and Chile (Berg et al., 2021). These authors stated that the activities of the illegal and informal mining activities are contributing extensively to environmental destruction, including deforestation, water pollution and the obliteration of critical ecosystems. Similarly, Scheidel et al. (2023) posited that these activities are severely impacting Indigenous communities through displacement, cultural disruptions, and conflicts over land. The influx of miners often brings diseases to isolated populations and creates zones of lawlessness marked by exploitation, poor working conditions, organised crime, and violence (Schwartz et al., 2021). Economically, these illegal mining operations have undermined the stability of many Latin American nations by promoting black market economies and resulted in significant revenue losses due to tax evasion and unregulated trade (Kurylo, 2024).

In light of the severe consequences of informal and organised illegal mining operations on the environment, many countries across the continent and other regions have enacted legislation and regulations to combat these activities (Hentschel et al., 2003). In Ghana, the Minerals and Mining Act (Act 703 of 2006) and its amendment (Act 995 of 2019) empower authorities to seize equipment and arrest offenders while imposing strict penalties (Asumda, 2022; Bansah et al., 2023). Farouk et al. (2023) stated that Nigeria's Minerals and Mining Act (2007) and Mining Regulations (2011) provide a comprehensive framework for licensing and penalties for informal

and illegal mining operations. The Democratic Republic of Congo (D.R.C.) enforces the Mining Code (Law No. 007/2002) and Mining Regulations (2003) to regulate and penalise illegal mining activities (Ngalula et al. 2023). Tanzania's Mining Act (2010) and the Natural Wealth and Resources (Permanent Sovereignty) Act (2017) emphasise licensing and sovereignty over natural resources (Rwiza et al., 2023). Zimbabwe combats illegal mining through the Mines and Minerals Act (Chapter 21:05) and the Environmental Management Act (Chapter 20:27), which focus on licensing and environmental protection (Matshusa et al., 2022). Joint regional and international efforts, such as the Kimberley Process Certification Scheme (KPCS) and the African Mining Vision (AMV), support transparent, equitable, and optimal exploitation of mineral resources while preventing the trade of illicit diamonds and enhancing governance in the mining sector (Gisore et al., 2015). In spite of these comprehensive regulations and legislation, curtailing these practices has stalled in many countries on the continent mainly due to pervasive corruption and weak governance that undermine enforcement, high profit margins and economic dependency (Towah et al., 2019). Asuamah (2023) further disclosed that cultural acceptance of organised illegal and informal mining operations and lack of awareness about its impacts, insufficient resources and technological limitations are hindering effective monitoring, persistent high global demand and complex supply chains that facilitate the entry of illegally mined minerals into legal markets, and the control of mining operations by organised crime and militant groups have elevated these activities instead of curtailing them.

1.2 Categorisations and Operationalisation of Organised Illegal Mining Activities in South Africa

Many scholarly literature and commentators, including Phala et al. (2019), William (2011), Khubani et al. (2022) and Madimu (2022), disclosed that there are levels of organised illegal and informal mining activities in South Africa and are strongly connected and supported by individuals, organisations and cooperatives of higher status in societies. (S2) depicts the levels of operations of illegal mining activities in the country.

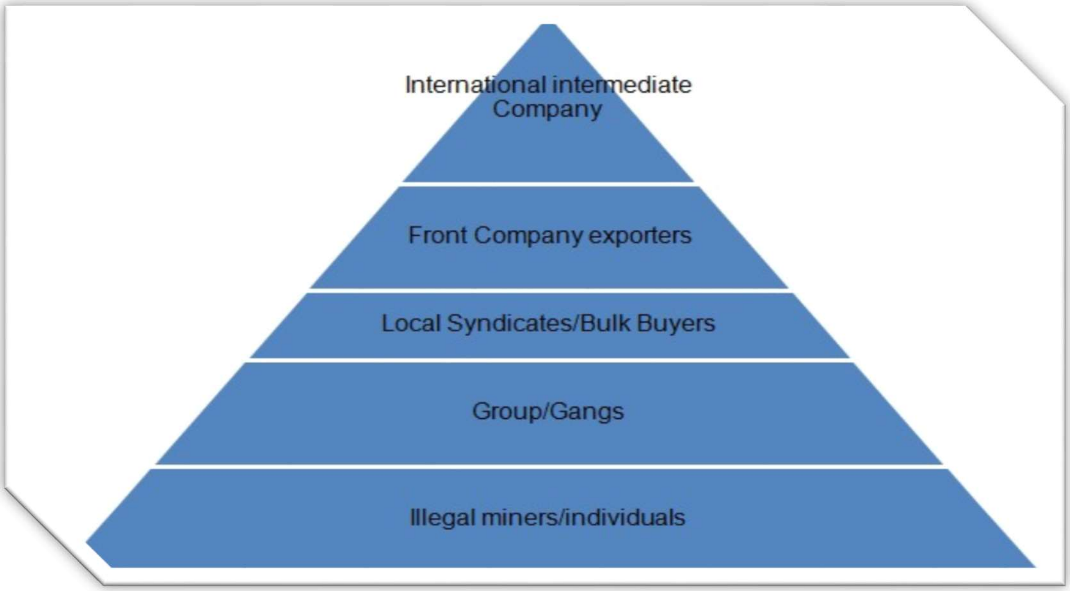

(S2): Levels of operationalisation of illegal mining activities. Source: South African Human Right Commission, 2013

The activities of organised illegal and informal mining operations in South Africa exist in five layers, according to the SACM (2017) report. The Minerals Council of South Africa (MCSA (2021) concurred that illegal mining and organised are interrelated. On most occasions, illegal mining operations globally are spearheaded by criminal syndicates. Brown (2017) further alluded that these illegal miners are mostly well-armed, equipped with explosives to carry out complex operations, lay ambushes, and provide security personnel to protect them against their rival groups of illegal miners. Phala et al. (2019) disclosed that vulnerable and undocumented migrants are conscripted by intermediaries and syndicates who will be promised financial rewards and improved lifestyles. The groups of individuals who serve as "foot soldiers or Zama-Zamas" will go secretive to engage in physical digging. These groups usually are at the bottom of these illicit activities (Gajigo et al., 2012). The second tier in the operational chain is the "gangs", whose responsibility is to ensure illegal miners' safety and security against rival groups (William, 2021). Furthermore, the gangs are usually community members or citizens who have in-depth knowledge of the area. Furthermore, this group is responsible for the recruitment and supplying of basic needs such as water, food, clothes, lighting, and other equipment required to carry out illegal activities (Mhlongo, 2023). The third layer within the structure is the "syndicates or bulk buyers", whose main task is to establish both local and international networks of buyers and transportation (Tite et al., 2019). Notably, their principal obligation is to ensure that the extracted minerals reach national and global markets through registered companies (Phala et al., 2017). The next level, which is the fourth layer, is the "Front Company Exporters". This layer of operation is individuals who are well-connected and up-to-date about the export of minerals on a large scale at large international markets (Debrah et al., 2014). Finally, at the apex of the syndicate are the

International Intermediaries whose main responsibility is to distribute the minerals to their respective countries (Chelin et al., 2021 and Yiridomoh, 2021).

1. 3. Environmental Problems Caused by Organised Illegal and Informal Mining Activities

1.3.1. Implication of Organised Illegal and Informal Mining on Water Resources in South Africa

Excavation and refinery operations of minerals require a high volume of water as every stage requires a large amount of water usage (Witchalls, 2022). For instance, dust lessening, moving soluble properties, sifting and separation processes and creating tailing dams for water management (Witchalls, 2022). Ochieng et al. (2010) disclosed that while each stage of the mining process has a bearing on water resources, significant damage is done when straying the mineral to remove airborne dust. This often leads to high water pollution levels and prevents water from being recycled (Ochieng et al., 2010). Similarly, Mononen et al. (2022) concurred that mining activities significantly affects the surrounding watercourses in different forms during the production phase. Worlanyo et al. (2021) expressed that discharged water from the mining centres is the biggest and most significant source of contamination to the surrounding stream. These authors argued that the wastewater is purposively discharged from the miners and controlled to flow through channels to nearby rivers, streams, lakes and dams, thereby contaminating the water with sulphur acid and ores. Furthermore, the alterations of land use due to the extraction operations also impact the hydrology of the area through changes in the levels of infiltration, overflow of water quantities and directions of water movement (Widana, 2019). Additionally, the clearing of vegetation often accelerates surface runoff and soil leaching, which eventually will increase the quantity of solid wastes that are carried to watercourses. The end result of these activities is that they will obscure and impact the quality of water locally, affecting the living conditions of aquatic organisms.

1.3.2. Implications of Mining on the Land Resources

Environmental problems associated with informal and organised illegal mining operations are changes that occur on the land, not only from digging and cutting open pits or holes but also the modifications that occur due to the infrastructure development of the environment (Witchalls, 2022). Many scholars, including Maphanga et al. (2023) and Dalu et al. (2017), highlight that organised illegal and informal mining in South Africa has severe impacts on the land, resulting in extensive environmental degradation, soil erosion and significant landscape alterations. These authors posited that approximately 14,000 hectares of land have been degraded in Gauteng province alone due to illegal mining. The affected communities and areas impacted by these activities experience soil erosion rates up to 20 times higher than unaffected communities (Maphanga et al., 2023). The landscape is further marred by dangerous open pits, sinkholes, and unstable ground, with over 6,000 abandoned mine sites in the country largely attributed to illegal mining (Mhlongo et al., 2019). Supporting this assertion, Mononem et al. (2022) expressed that mining required infrastructure such as mining pits, side-stone heaps, mass soil removal, tailing

areas, landfill sites, buildings, and infrastructures, such as roads, water and electricity lines which involve direct destruction of habitats and displacement of flora and fauna. Widana (2019) posited that the vast spaces where this mining occurs have contributed to massive destruction of vegetation, desertification, soil erosion and changes in soil structure in South Africa and beyond. These changes have led to the loss of biodiversity and land degradation in the many provinces of South Africa, especially Gauteng, KwaZulu-Natal, Northwest and Mpumalanga provinces (Worlanyo et al., 2022).

1.3.3. Mining Activities and Gas Emission

The destruction of forests and degradation of land as a result of mining activities have led to massive emissions of carbon dioxide and other harmful gases into the atmosphere (Witchalls, 2022). The extraction sector emits between "1.9 and 5.1 gigatons of carbon dioxide (CO_2) equivalent (CO_2e) of GHG emissions annually" (Bagli et al., 2017: 37). Olufemi et al. (2018) disclosed that the majority of emissions from the mining sector originate from coal. Olufemi et al. (2018) postulate that methane released during coal mining ranges from 1.5 to 4.6 gigatons, mainly in underground mining operations. Similarly, Obeng et al. (2019) expressed that during extraction activities, numerous harmful gases, notably CO_2, methane, nitrogen oxide and other heat-trapping gases, are released, which stay in the atmosphere for many years. The impact of the extractions and processing industry in a locality is that the emission of coal-related air pollution remains extremely high and prone to different health hazards (Pone et al. 2007). South Africa has one of the highest concentrations of these toxic, dangerous substances in the atmosphere and is recorded to be among the filthiest air globally (Maya et al. 2015; Munnik et al. 2010). "This air pollution hotspot was declared by the Department of Environmental Affairs a Highveld priority area in terms of the National Environmental Management: Air Quality Act 39 of 2004 Department of Environmental Affairs" (D.E.A. &MR 2014: 143; Munnik et al. 2010). The impact of coal mining is not only restricted to air pollution but has extended implications for global warming. (Lockwood et al. 2009; Berrill et al. 2016). For example, CO_2 emission has significantly increased over the last five decades and persistently increases every passing year even though the global community are trying to curtail it. While it is acknowledged that CO_2 is the largest cause of global warming, new studies have proved that methane is 21 times more effective since this gas is more destructive to the ozone layer than CO_2 (Mar et al., 2022). Table 3 summarises the impact of mining on the environment

Supplementary Table (S3): Summary of impacts of mining on land, water and air

Land Resources	Water Resources	Emission of greenhouse gases
Changes to land, topography and landscape	Changes in hydrology	Increase of air pollution in the atmosphere
Erosion and loss of biodiversity	Ground and surface water impacts	The concentration of carbon dioxide and nitrogen in the atmosphere

Waste rock and tailings	Changes in water quality (e.g. clouding, odour and colour)	Decrease in air quality
Alteration of soil profiles and risks of contamination	Controlled wastewater discharges and their cumulative impacts	Increase of airborne diseases
Habitat changes, fragmentation and loss	Increasing water scarcity in arid and semi-dried areas	Climate change variability and global warming
Loss of vegetation and deforestation	Risks of contamination, acid mine drainage, dam accidents, dewatering	Radiation dust, small and noise pollution
It affects agricultural production, leading to food insecurity	Outbreaks of communicable diseases, conflicts and water insecurity	Respiration implication for humans and animals

Source: Department of Forestry, Fisheries and the Environment, 2015

1.4. Legislation and Policies Guidance for Informal Mining Activities in South Africa

The Department of Environment and Forestry (DEF) and the Mineral and Petroleum Resources Development (MPRD) are statutory bodies established under the 1996 Constitution of South Africa to regulate, coordinate, promote, safeguard environmental protection, enforce regulations, prevent and control pollution, support sustainable development, enforce clean environment and issue mining permit (van der Bank et al., 2020). Furthermore, these departments are to ensure that environment the is safe and not harmful to people's health and welfare, flora and fauna, as well as biodiversity are protected (van der Bank et al., 2020). According to the Mines, Minerals and Environmental Regulation (MMER) of 1999 under the DEF, any company or individuals wishing to undertake any mining operation in South Africa, particularly the commercial mining activities that have a significant impact on the environment, must obtain written permission from DEF and MPRP after undergoing an Environmental Impact Assessment (E.I.A.) a key requirement under National Environmental and Management Act of 1998 (NEMA (du Plessis, 2005). The Regulations in terms of Chapter 5 of the National Environmental Management Act (NEMA), 1998 (Act 107 of 1998) define reconnaissance, prospecting, mining or retention operations as provided for in the MPRDA as a listed activity that requires a Basic Assessment Report (B.A.R.) to be undertaken" (Ndlazi, 2021: 224). Under B.A.R., an E.I.A. evaluation is required; this involves a systematic investigation of elements in the environment that the planned development or proposed project will negatively impact. The assessment must provide a forecast for the developer and planning authorities to expect implications and degree of damage to the environment (Ridl et al., 2010; Swart et al., 2022). In terms of Section 39(2) of the Mineral and Petroleum Resources Development Act, 2002 (Act 28 of 2002) "any person who applies for reconnaissance permission, prospecting right or mining permit must submit an Environmental Management Plan (E.M.P.) as prescribed to the Regional Manager concerned for his approval" (Swart et al., 2022: 11). Furthermore, Section 5(4), E.M.P. stated that "no person will undertake reconnaissance operations or any other activity without an approved E.M.P., right, permit or permission or without notifying the landowner (Swart et al., 2022: 70). Additionally, in terms of Section 107 of National Environmental Management Act

(NEMA 1998) of the Minister must consult each department charged with the management of any legislation related to any issues affecting the environment before approving (Ledwaba, 2017). Figure 2 simplifies the framework for mine management in South Africa.

Supplementary Figure (S.2): The framework of mine management in South Africa

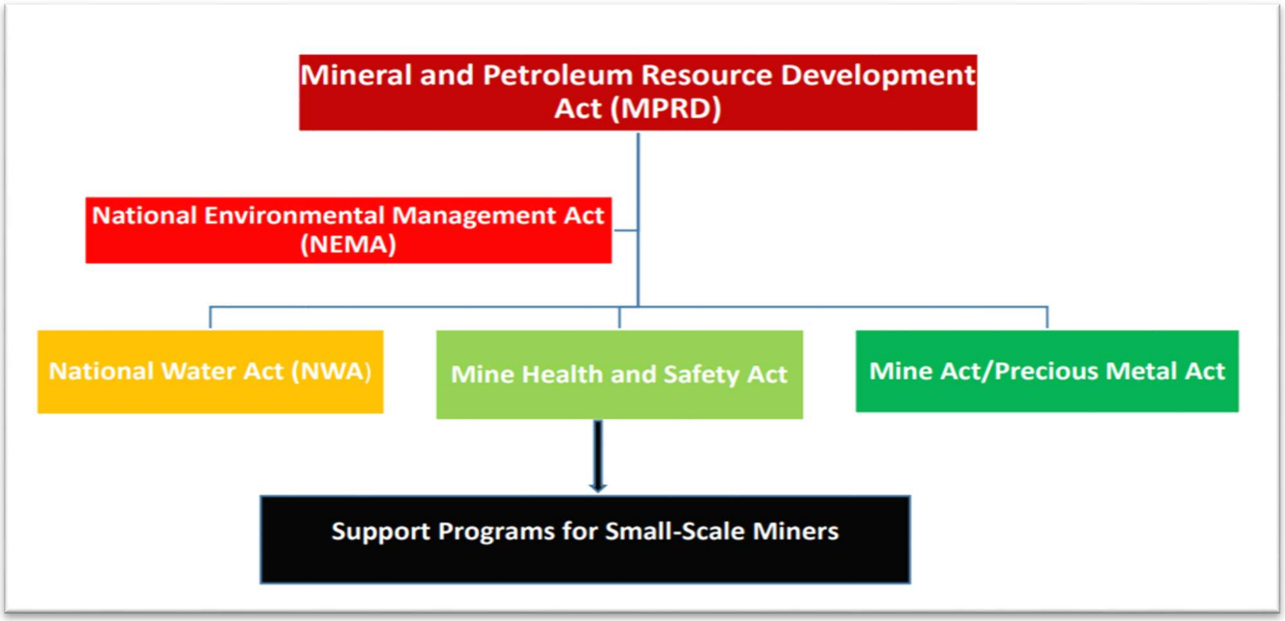

Source: Department of Mineral Resources, 2019

While these policies and regulations guiding mining operations are well defined, the environmental implications linked to these activities have often been ignored and worsening. Numerous literature, including Mhangara et al. (2020) and Ncube-Phiri et al. (2015), disclosed that most often, regarded as mere procedural and 'tick box' processes. Nevertheless, given the

exponential rise of this sector and the haphazard nature of their management, informal and organised illegal mining activities pose a significant threat to water and land resources and the flora and fauna wealth of the country a review of the entire processes are required (Agwa-Ejon et al., 2018). There is the need to address the challenges of this sector by enforcing the legislation, regulations and strategies envisaged by the government in the NEMA regulations of 1998 and Mineral and Petroleum Resources Development Act (Act 28 of 2002) (Ledwaba, 2017).

2. The Study Area

Supplementary Figure (S3): Map of study site

While this study looked into the impact of informal and organised illegal mining activities in South Africa as a whole, data collections for this study was restricted to selected communities in Gauteng and Free State provinces. Specifically, it is from Benoni, Boksburg, Daveyton, Germiston, Krugersdorp and Welkom. Figure 5 depicts the study area.

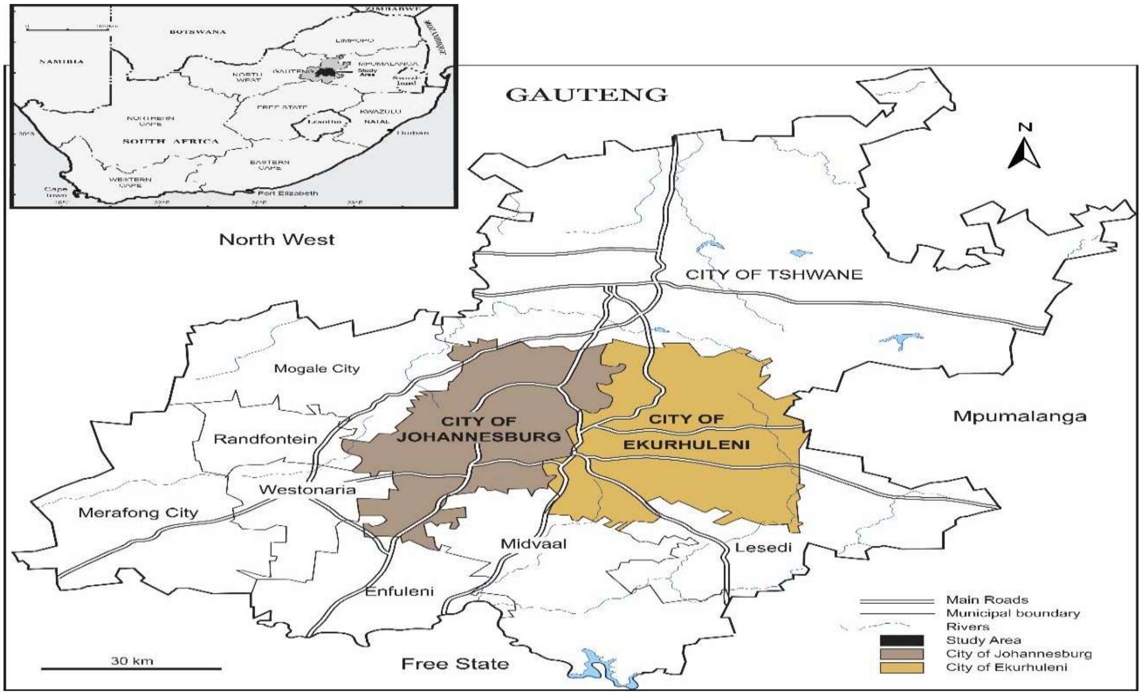

Figure 3: The map of the study site in Gauteng: Source: The Department of Cartography and Geographical Information System section, University of Witwatersrand, 2023

The choice of Gauteng and Free State provinces were based on the grounds that the provinces are the richest in terms of mineral deposits in the country, and more than 60% of the population in the provinces earns their livelihoods directly and indirectly from mining activities (Cairncross et al., 2012). The provinces have a variety of minerals, ranging from gold, diamond, iron ore, platinum, manganese, chromium, copper and titanium (Southwood et al., 2017). Gauteng and Free State contribute over 50% of all gold produced in the country (Cairncross, 2012). The mining sites are spread within a chain of gold fields that are, in anti-clockwise order from the north-east: Evander,

over a gap of approximately 60 km across a basement granite to the contiguous East Rand, Central Rand, West Rand, South Deeps, Western Areas and Carletonville gold field which represent around 150 km (Ngcofe et al., 2014). The Klerksdorp gold field is located about 80 km to the southeast of Klerksdorp. While mining remained the main source of revenue for the Gauteng Province, the sector has lost its reputation as a once large-scale mega-mining hub due to the depletion of the minerals and the invasion of Zama-Zamas (Trango and Bobbins, 2015). Currently, these provinces, have the highest concentration of abandoned mines, huge migrant population seeking economic opportunities, organised criminal networks supporting illegal mining operations and proximity to urban centres facilitating the trading and movement of illicitly mined gold. Similarly, the province has a significant percentage of informal and mining operating as survivalist feeding themselves and their families (Mothetha, 2009). These complex and interwoven factors present perfect grounds as a case study into the illegal mining activities in South Africa.

3. Supplementary Results

Additional detailed results and supporting data exploring the implications of organised and informal mining activities on the environment are provided in the supplementary materials.

3.1. Primary and underlying drivers to illegal and informal mining in South Africa

A community leader who was once an illegal miner disclosed this when approached for an interview. He stated that:

> "The people with whom he was working were those very desperate, self-organised Zama-Zamas, who were of the view that illegal mining activities were means to work for themselves to be self-independent and survive in the challenging economic environment, and who think that they are doing activities so as to avoid criminality, to avoid being the kinds of people who would abduct other people's resources or wealth or rob others or even become full-scale arm robbers" Per.com 2024 A.

These sentiments were echoed by another respondent from the Gauteng Department of Mineral Resource who was engaged in an interview. He disclosed that:

> "Regional poverty largely accounts for the supply of illegal miners, predominantly from neighbouring countries, specifically Lesotho, Swaziland, Malawi, Zimbabwe and Mozambique. According to this interviewee, due to economic challenges in their home countries, they flee to South Africa, and many become victims to criminal syndicates who traffic refugee labour into operational and abandoned mines" Per.com 2024 B.

A community leader who was once an illegal miner disclosed this when approached for an interview. He stated that:

"The people with whom he was working were those very desperate, self-organised Zama-Zamas, who were of the view that illegal mining activities were means to work for themselves to be self-independent and survive in the challenging economic environment, and who think that they are doing activities so as to avoid criminality, to avoid being the kinds of people who would abduct other people's resources or wealth or rob others or even become full-scale arm robbers" Per.com 2024 C.

3.2. Impacts of illegal mining operations on the environment

The implications of illegal mining were investigated extensively with respondents. The following are the views of some respondents. A lecturer in mining at the Department of Mining and Engineering at the University of Witwatersrand stated in an interview that:

"The informal illegal mining activities contribute to water pollution, including metal contamination, increased sediment levels in streams, and acid mine drainage. Pollutants released from processing plants, tailing ponds, underground mines, waste-disposal areas, active or abandoned surface or haulage roads, etc., act as the top sources of water pollution. Sediments released through soil erosion cause siltation or the smothering of stream beds. It adversely impacts irrigation, swimming, fishing, domestic water supply, and other activities dependent on such water bodies " Per.com 2024 D.

Similar sentiments were shared by a community member who was engaged in an interview. He disclosed that:

"The mining activities create landscape blots like open pits and piles of waste rocks due to mining operations that often lead to the physical destruction of the land at the mining site. These disruptions contribute to the deterioration of the area's flora and fauna, degradation and soil erosion. He further postulates that many surface features present before mining activities cannot be replaced after the process has ended. The removal of soil layers and deep underground digging often destabilize the land and render the land unusable" Per.com 2024 1E.

Another respondent from the community also mentioned that:

"Mining operations routinely modify the surrounding landscape by exposing previously undisturbed earthen materials. Erosion of exposed soils extracted mineral ores, tailings, and fine material in waste rock piles can result in substantial sediment loading to surface waters and drainage ways. In addition, spills and leaks of hazardous materials and the deposition of contaminated wind" Per.com 2024 F.

We sought respondents' views through face-to-face interviews regarding air pollution as a consequence of informal and illegal mining undertakings. An employee from the Gauteng Department of Environment and Forestry said this when She was approached:

"There are periods of air pollution as the concentration of these chemicals in the air sometimes becomes high, resulting in a gas fuse and other problems. In addition, there are

events of exposures to chemicals such as cyanide, harmful materials and others by both workers and people close to the surrounding communities" Per.com 2024 G.

3.3. Strategies to address illegal mining operations in South Africa

A Senior Lecturer shared a similar view in the Department of Mining Engineering at the University of Witwatersrand. The interviewee stated that:

> "The country must invest in research and development of green mining technology (GMT). According to this respondent, the informal mining sector needs proper research and development to ensure that the sector copes with the ever-changing trends of today's sustainability and "green friendly" operations. State institutions collecting funding and allowing that funding to be dispersed into R.O.D. funds for green mining can be one way to positively impact the environment before and after mining projects. By pushing the envelope and never letting the future slip too far from reach, staying ahead can prevent unnecessary waste in the sense of less reusable materials, better efficiency and a better understanding of the sector" Per.com 2024 H.

A member of a community in the Krugersdorp in the Gauteng Province proposed during the one-on-one interview that:

> "Green mining education, including technical, environmental training and information, will increase expertise, capacity and know-how to improve informal mining and minimise environmental degradation that comes with mining activities. In South Africa today, the youth and women face multiple, interconnected barriers that restrict their ability to own mining licences and permits to undertake legitimate mining activities. Resource allocation gaps make it extremely difficult to be inculcated into the mainstream economy and live a meaningful life " Per.com 2024 I.

Supplementary: References

Ackerman, M., van der Waldt, G., & Botha, D. (2018). Mitigating the socio-economic consequences of mine closure. *Journal of the Southern African Institute of Mining and Metallurgy, 118*(4), 439-445. https://doi.org/10.17159/2411-9717/2018/v118n4a14.

Afriye, K., Abass, K., Frempong, F., Arthur, B., & Gyasi, R. M. (2023). The dynamics and livelihood implications of illegal mining in Ghana: A critical assessment. *Geographical Research, 61*(1), 32-43. DOI: https://doi.org/10.1111/1745-5871.12573

Agwa-Ejon, J., & Pradhan, A. (2018). Life cycle impact assessment of artisanal sandstone mining on the environment and health of mine workers. *Environmental Impact Assessment Review, 72*(2018), 71-78. https://doi.org/10.1016/j.eiar.2018.05.005

Andrew, N. (2015). Digging for survival and/or justice? The drivers of illegal mining activities in Western Ghana. *Africa Today, 62*(2), 2-24. DOI: 10.2979/africatoday.62.2.3

Asore, M., Mpobi, R. K., Morgan, A. K., Apoanaba, T. A., Katey, D., Ampofo, S. T., . . . Appiah, D. O. (2023). Is illegal mining socio-politically entrenched? An opinion piece on the interaction between formal politics and chief dominance in mineral governance and its influence on fighting Galamsay in Ghana. *GeoJournal, 88*(2023), 1953-1963. doi: 10.1007/s10708-022-10725-1

Asumda, D., Situma, D. P., Muigua, K., & Issahaku, S. (2022). The available legal regime and the use of mercury for informal gold mining in Ghana. *International Journal of Scientific, 9*(4), 3-7. DOI: 10.32628/IJSRSET22945

Berg, R., Ziermer, H., & Kohan, A. (2021). *A closer look at Colombia's illegal artisanal and informal mining.* Colombia: Centre for Strategic and International Studies. https://www.csis.org/analysis/closer-look-colombias-illegal-artisanal-and-small-scale-mining

Berrill, P., Arvesen, A., Scholz, Y., Gils, H. C., & Hertwich, E. (2016). Environmental impacts of high penetration energy scenarios for Europe. *Environmental Research Letters, 11*(2016), 2-11. DOI: 10.1088/1748-9326/11/1/014012

Burger, D., & Saunders, K. (2019). *Understanding and mitigation lead exposure in Kabwe: A one health approach.* Pretoria: Southern Africa Institute for Policy and Research. saipar.org/wp-content/uploads/2020/03/Saunders.Burga_.CCZ_-1.pdf

Cairncross. (2012). *Southern African minerals photo-essay.* Tshwane: City of Tshwane Metropolitan Municipality. DOI: 10.1080/00357529.2012.709172

Dalu, T. B., Wasserman, R. J., & Dalu, T. (2017). A call to halt destructive, illegal mining in Zimbabwe. *South African Journal of Science, 113*((11/12)), 1-3. http://dx.doi.org/10.17159/sajs.2017/a0242

Darko, G., Obiri-Yeboah, S., Takyi, S. A., Amposah, O., Borquaye, L. S., Amposah, L. O., & Fosu-Mensah, B. Y. (2022). Urbanising with or without nature: Pollution effects of human activities on water quality of major rivers that drawn the Kumasi Metropolis of Ghana. *Environmental Monitoring and Assessment, 194*(38), 10. DOI: 10.1007/s10661-021-09686-8

Day, M., Gumbo, D., Moombe, K. B., Wijaya, A., & Sunderland, T. (2014). *Zambia country profile monitoring reporting and verification for REDD+.* Bogor, Indonesia: Centre for International Forestry Research. https://doi.org/10.17528/cifor/004932

Debrah, A., Watson, I., & Quansah, D. P. (2014). Comparison between artisanal and informal mining in Ghana and South Africa: Lessons learnt and ways forward. *Journal of the South African Institute, 14*(11), 913. Metallurgy, 2014. ISSN 2225-6253.

du Plessis, C. (2005). Action for Sustainability: Preparing an African plan for sustainable construction. *Building Research and Information, 33*(5),1-11.Email: cdupless@csir.co.za

Farouk, A. U., Maigoshi, Z. S., & Jibril, R. S. (2023). Enhancing revenue generation through mining activities in Nigeria. *International Journal of Corporate Reporting Taxation and Finance, 1*(1), 3-9. Website: www.abupress.com.ng

Frederiksen, T. (2019). Political settlements, the mining industry and corporate social responsibility in developing countries. *Extractive Industries and Society, 6*(1), 162-170. https://doi.org/10.1016/j.exis.2018.07.007

Gajigo, O., Mutambatsere, E., & Ndiaye, G. (2012). *Gold mining in Africa: Maximising economic returns for countries.* Tunis: African Development Bank. http:/www.afdb.org/

Gisore, R., & Matina, Z. (2015). *Sustainable mining in Africa: Standards as essential catalysts.* Nairobi, Kenya: ARSO Central Secretariat. https://www.arso-oran.org/wp-content/uploads/2014/09/Sustainable-Mining-in-Africa-Standards-as-Catalysts.pdf

Hatu, R. A. (2016). Socio-economic conditions in the illegal gold miners Tulabolo village, Gorontalo - in Indonesia. *Asian Journal of Applied Sciences, 9*(2016), 97-100. DOI: 10.3923/ajaps.2016.97.105

Jongwe, R., & Mhlanga, D. (2023). Livelihood activities in post-independent Africa: A close look at the impact of Chikorokoza illegal mining on the educational system in Zimbabwe. *Advances in African Economic, Social and Political Development*, 70-79. DOI: 10.1007/978-3-031-30541-2_7

Khubani, J., & Kulkami, S. (2022). "Group search optimiser-based neural network for EEG-based emotion recognition", 2022. *Intelligence and Computing (ICAAIC)*, (pp. 187-194). ICAAIC. DOI: 10.1109/ICAAIC53929.2022.9792986

Kurylo, B. (2024). *The environmental impact of illegal mining in Latin America.* Earth.Org. https://earth.org/the-environmental-impact-of-illegal-mining-in-latin-america/

Kyere-Boateng, R., & Marek, M. V. (2021). Analysis of the social-ecological causes of deforestation and forest degradation in Ghana: Application of DPSIR framework. *Forest, 2021*(2), 3-9. https://doi.org/10.3390/f12040409

Lockwood, A. H., Fean, M. D., Welker-Hood, K., Molly, R., & Gottlieb, B. (2009). *Coal's assault on human health.* Washington DC: International Physician. www.psr.org/coalreport.

Madimu, T. (2022). Illegal gold mining and the every day in post-apartheid South Africa. *Review of African and Political Economy, 49*(16), 10-15. DOI: 10.1080/03056244.2022.2027750

Mancini, L., & Sala, S. (2018). Social impact assessment in the mining sector: Review and comparison indicators framework. *Resource Policy, 57*(2018), 98-111. https://doi.org/10.1016/j.resourpol.2018.02.002

Mar, K. A., Unger, C., Walderdorff, L., & Butler, T. (2022). Beyond CO2 equivalence: The impacts of methane on climate, ecosystem and health. *Environmental Science & Policy, 134*(2022), 127-136. https://doi.org/10.1016/j.envsci.2022.03.027

Maya, M., Musekiwa, C., Mthembi, P., & Crowley, M. (2015). Remote sensing and geochemistry techniques for assessment of coal mining pollution Emalahleni (Witbank), Mpumalanga. *South African Journal of Geomatics, 4*(2), 3-10. DOI: 10.4314/sajg.v4i2.9

Mazikana, T. A. (2022). *Assessing the impact of illegal mining on the environment: A case of Ward 59 of Mt Darwin South District.* Johannesburg: SSRN. http://dx.doi.org/10.2139/ssrn.4154451

McFarland, C., & Talos, C. (2018). *The illicit cryptocurrency mining threat.* Cyber Threat Alliance https://www.cyberthreatalliance.org/wp-content/uploads/2018/09/CTA-Illicit-CryptoMining-Whitepaper.pdf

Mhangara, P., Tsoeleng, L. T., & Mapurisa, W. (2020). *Monitoring the development of artisanal mines in South Africa.* Pretoria: South African Institute of Mines and Metallurgy. https://doi.org/10.17159/2411-9717/938/2020

Mononem, T., Kotilainen, J., Kivinen, S., & Leino, J. (2022). *Social and environmental impacts of mining activities in the E.U.* Finland: University of Eastern Finland. http://www.europarl.europa.eu/supporting-analyses

Mothetha. (2021). *Mineral and mining development study of the Molemole Local Municipality, Limpopo.* Polokwane: Council for Geoscience, Limpopo Unit. http://www.molemole.gov.za/docs/mine/MINERAL%20AND%20MINING%20DEVELOPMENT%20STUDY.pdf

Moyo, T., Chitaka, T., Lotter, A., Schenck, C., & Petersen, J. (2022). Urban mining versus artisanal and informal mining (A.S.M.): An interrogation of their contribution to sustainable livelihoods in Sub-Saharan Africa. *The Extractive Industries and Society, 12*(2022), 3-6. https://doi.org/10.1016/j.exis.2022.101173

Mulenga, F. K., & Dikgwatlhe, P. (2023). Perception of local communities regarding the impacts of mining on employment and economic activities in South Africa. *Resources Policy, 80*(1), 3-10. DOI: 10.1016/j.resourpol.2022.103138

Munnik, V. (2010). *The social and environmental consequences of coal mining in South Africa: A case study.* Pretoria: Environmental Monitoring Group. https://www.researchgate.net/publication/364742370

Ncube-Phiri, S., Ncube, A., Mucherera, I., & Ncube, K. (2015). Artisanal informal mining: Potential ecological in Mzingwane District, Zimbabwe. *Jamba, 7*(1), 158 doi: 10.1080/03736245.2020.1823875

Ndlazi, S. (2021). "Alone in the dark" is how the current legal regime for mining and minerals continues to fail artisanal and informal in South Africa. *Law, Democracy and Development, 25*(8), 222-225. https://orcid.org/0000-0001-8717-6815

Ngcofe, L., & Cole, D. I. (2014). The distribution of the economic mineral resource potential in the Western Cape Province. *South African Journal of Science, 110*(2), 5-6. http://dx.doi.org/10.1590/sajs.2014/a0045

Nti, E. K., Kranjac-Berisavljevic, G., Doke, D. A., Wongnaa, C. A., Attafuah, E. E., & Gyan, M. A. (2023). The impact of artisanal gold mining on the sustainability of Ghana's river basin: The case of Pra basin. *Environmental and Sustainability Indicators, 19*(2023), 6-12. https://doi.org/10.1016/j.envc.2023.100804

Ofosu, G., & Sarpong, D. (2023). Defying the gloom: In search of the golden practices of informal mining operation. *Environmental Science & Policy, 139*(2023), 62-70 https://doi.org/10.1016/j.envsci.2022.10.013

Olufemi, C. A., Bello, P. O., & Mji, A. (2018). *Conflict implications of coal mining and environmental pollution in South Africa: Lessons from Niger Delta, Nigeria.* Pretoria: Faculty of Humanities, Tshwane University of Technology. https://www.ajol.info/index.php/ajcr/article/view/175827

Oruonye, E. D., Musa, D. G., & Ahmed, Y. M. (2023). Challenges of enforcement of government ban of illegal mining in Taraba State Nigeria. *International Journal of Social Science and Humanities Research, 6*(11), 8-10. DOI: https://doi.org/10.5281/zenodo.10090348

Pone, N., Hein, A. A., Stracher, G. B., & Annegarn, H. J. (2007). The spontaneous combustion of coal and its by-products in Witbank Sasolburg coalfields of South Africa. *International Journal of Coal Geology, 72*(2), 124-125. 10.1016/j.coal.2007.01.001

Ridl, J., & Couzens, E. D. (2010). Misplacing NEMA is a consideration of some problematic aspects of South Africa's new E.I.A. regulations. *P.E.R, 13*(5), 2-5. DOI: 10.4314/pelj.v13i5.65052

Ruiza, M. J., Focus, E., Bayou, J., Kimaro, J. M., Keleinke, M., Lyasenga, T. J., . . . Marwa, J. (2023). Artisanal and informal mining in Tanzania and health implications: A policy perspective. *Heliyon, 9*(4), 3-10. doi: 10.1016/j.heliyon.2023.e14616

Schwartz, F. W., Lee, S., & Darrah, T. H. (2021). A review of the scope of artisanal and informal mining worldwide, poverty and associated health impacts. *GeoHealth, 5*(2021), 3 DOI: 10.1029/2020GH000325

Suglo, P., Effah, P., Acheampong, A. A., Sunkari, R., & Yeboah, A. (2021). Effects of illegal mining on the environment, economy and agricultural productivity. *Biochemistry and Molecular Biology, 6*(4), 79-91. http://www.sciencepg.com/journal/bmb

Soe, P. S., Kyaw, W. T., Arizono, K., Ishiashi, Y., & Agusa, T. (2022). Mercury pollution from artisanal and informal gold mining in Myanmar and other Southeast Asian countries. *International Journal of Environmental Research and Public Health, 19*(10), 6290. https://doi.org/10.3390/ijerph19106290

Southwood, M., & Cairncross, B. (2017). The minerals of Parabora, Limpopo Province, South Africa. *Rock & Minerals, 92*(5), 426-453. DOI: 10.1080/00357529.2017.1331398

Swart, C., Dalasile, S., Louw, S., Armitage, M., & Clark, B. (2022). *Draft scoping report: Environmental Impact Assessment for prospecting rights with bulk sampling for Kaolin Silica Xwenna.* Cape Town: Anchor Environmental Consultants. info@anchorenvironmental.co.za

Towah, W. D. (2019). *The impact of good governance and stability on sustainable development in Ghana.* Walden: Walden University Scholarworks. https://enactafrica.org/enact-observer/south-africas-illegal-mining-conundrum

Trango, G., & Bobbins, K. (2015). Gold mining exploits and legacies of Johannesburg's mining landscape. *Scenario Journal, 2015*(1), 2-10. https://scenariojournal.com/article/gold-mining-exploits/ (scenariojournal.com)

van der Bank, M., & Karsten, J. (2020). Climate change and South Africa: A critical analysis of the Earthlife Johannesburg and another V Minister of Energy and others 65662/16 (2017) case and drive for concrete climate practices. *Air, Soil and Water Research, 13*(2020), 1-11. https://doi.org/10.1177/1178622119885372

Verweijen, J., Schouten, P., & Simpson, F. (2022). *Conservation, conflict and semi-industrial mining: The case of Eastern D.R.C.* Antwerp, Belgium: Institute of Development Studies. University of Antwerp. DOI: 10.13140/RG.2.2.29462.42566

Widana, A. (2019). The impacts of the mining industry: Socio-economics and political impacts. *Electronic Journal, 2019*(10), 10-12. DOI: 10.2139/ssrn.3423562

William's, T. G. (2019). *Illegal mining's effects on the sustainability of a South African gold mine.* Mafiken: School of Businesss and Governance, North-West University. https://Orcid.org/0000-0002-1974-972X

Worlanyo, A. S., Alhassan, S. I., & Jiangfeng. (2022). The impacts of gold mining on the welfare of local farmers in Asutifi-North District in Ghana: A quantitative and multi-dimensional approach. *Resources Policy, 75*(2022), 5-10. DOI: 10.1016/j.resourpol.2021.102458

Yiridomoh, G. Y., Caldwell, W., Siakwah, P., & Krachling, P. (2021). "Illegal" gold mining operations in Ghana: Implication for climate-smart agriculture in Northwestern Ghana. *Frontiers in Sustainable Food Systems, 5*(2021), 5-15. https://doi.org/10.3389/fsufs.2021.745317
